# Mobile phone-delivered reminders and incentives to improve childhood immunisation coverage and timeliness in Kenya (M-SIMU): a cluster randomised controlled trial

**DOI:** 10.1016/S2214-109X(17)30072-4

**Published:** 2017-03-11

**Authors:** Dustin G Gibson, Benard Ochieng, E Wangeci Kagucia, Joyce Were, Kyla Hayford, Lawrence H Moulton, Orin S Levine, Frank Odhiambo, Katherine L O'Brien, Daniel R Feikin

**Affiliations:** a International Vaccine Access Center, Department of International Health, Johns Hopkins Bloomberg School of Public Health, Baltimore, MD, USA; b Department of International Health, Johns Hopkins Bloomberg School of Public Health, Baltimore, MD, USA; c Kenya Medical Research Institute/Centers for Disease Control and Prevention Public Health and Research Collaboration, Kisumu, Kenya

## Abstract

**Background:**

As mobile phone access continues to expand globally, opportunities exist to leverage these technologies to support demand for immunisation services and improve vaccine coverage. We aimed to assess whether short message service (SMS) reminders and monetary incentives can improve immunisation uptake in Kenya.

**Methods:**

In this cluster-randomised controlled trial, villages were randomly and evenly allocated to four groups: control, SMS only, SMS plus a 75 Kenya Shilling (KES) incentive, and SMS plus 200 KES (85 KES = USD$1). Caregivers were eligible if they had a child younger than 5 weeks who had not yet received a first dose of pentavalent vaccine. Participants in the intervention groups received SMS reminders before scheduled pentavalent and measles immunisation visits. Participants in incentive groups, additionally, received money if their child was timely immunised (immunisation within 2 weeks of the due date). Caregivers and interviewers were not masked. The proportion of fully immunised children (receiving BCG, three doses of polio vaccine, three doses of pentavalent vaccine, and measles vaccine) by 12 months of age constituted the primary outcome and was analysed with log-binomial regression and General Estimating Equations to account for correlation within clusters. This trial is registered with ClinicalTrials.gov, number NCT01878435.

**Findings:**

Between Oct 14, 2013, and Oct 17, 2014, we enrolled 2018 caregivers and their infants from 152 villages into the following four groups: control (n=489), SMS only (n=476), SMS plus 75 KES (n=562), and SMS plus 200 KES (n=491). Overall, 1375 (86%) of 1600 children who were successfully followed up achieved the primary outcome, full immunisation by 12 months of age (296 [82%] of 360 control participants, 332 [86%] of 388 SMS only participants, 383 [86%] of 446 SMS plus 75 KES participants, and 364 [90%] of 406 SMS plus 200 KES participants). Children in the SMS plus 200 KES group were significantly more likely to achieve full immunisation at 12 months of age (relative risk 1·09, 95% CI 1·02–1·16, p=0·014) than children in the control group.

**Interpretation:**

In a setting with high baseline immunisation coverage levels, SMS reminders coupled with incentives significantly improved immunisation coverage and timeliness. Given that global immunisation coverage levels have stagnated around 85%, the use of incentives might be one option to reach the remaining 15%.

**Funding:**

Bill & Melinda Gates Foundation.

## Introduction

Annually, immunisation programmes are estimated to save more than 2·5 million lives worldwide.^1^ However, approximately 18·7 million and 20·1 million children do not receive the third dose of diphtheria, tetanus toxoid, and pertussis-containing antigens (DTP3) or measles vaccine, respectively. Global coverage levels have stalled at about 85%.^2^ Innovative interventions that address the remaining 15% (ie, the last mile) are needed because this subpopulation represents a group of individuals at disproportionate risk of disease.

The decade 2010–2019 has been designated the Decade of Vaccines, with a renewed focus on improving immunisation coverage by major international groups and the development of the Global Vaccine Action Plan (GVAP).^3^ A key component of GVAP is the recognition that both supply-side and demand-side deficiencies need to be addressed to achieve universal immunisation.^4^ Efforts to make vaccines accessable to children even in the most remote places have made great progress. Yet, children remain undervaccinated, which might be, in part, because of residual demand-side constraints.

As mobile phone access and ownership continue to become more common worldwide,^5^ opportunities exist to leverage mobile-health (mHealth) technologies to target demand-side barriers, such as forgetting vaccination appointments, not knowing the vaccine schedule, or incurring transportation costs, to improve immunisation uptake. One of the more commonly deployed demand-side mHealth interventions is short message service (SMS), or text message, reminders. SMS reminders significantly improve both health-seeking behaviours and outcomes in sub-Saharan Africa^6^, ^7^, ^8^, ^9^, ^10^, ^11^ and immunisation uptake in the USA.^12^, ^13^ However, the evidence that these approaches increase immunisation coverage and timeliness in Africa and low-income and middle-income countries (LMICs) is insufficient and has predominantly focused only on timely pentavalent (DTP, *Haemophilus influenzae* type B [Hib] and Hepatitis B) vaccination.^14^, ^15^, ^16^, ^17^, ^18^, ^19^

**Research in context**

**Evidence before this study**

We searched Scopus, Embase, PubMed, Web of Science, Global Health, and Cochrane Library database for existing evidence of SMS reminders or monetary incentives to improve immunisation coverage in low-income countries from Jan 1, 1990, to Sept 30, 2013. We used the following search terms: “text messag*”, “short message service”, “SMS” “information communication technology”, “ICT”, “text reminder”, “incentive”, “money”, “payment”, “cash”, “conditional transfer”, “conditional cash transfer”, “CCT”, “voucher”, “subsidy”, “coupon”, “immunization”, “immunisation”, and “vaccination”. We found no examples of randomised controlled trials that assessed the efficacy of SMS reminders to improve immunisation coverage or immunisation timeliness in low-income and middle-income countries. For incentives, we found one cluster-randomised controlled trial from rural India showing that providing caregivers with lentils if their children received diphtheria, tetanus toxoid, and pertussis vaccination significantly improved immunisation coverage in children when measured in children aged 1–3 years old. We found no studies that used incentives in the form of cash or mobile-money to improve either immunisation coverage or timeliness.

**Added value of this study**

To our knowledge, this is the first randomised controlled trial that has assessed the effect of SMS reminders, with or without monetary incentives, to improve the proportion of fully immunised children. A recently published randomised controlled trial from Zimbabwe that enrolled children presenting to the clinic found significant effects of SMS reminders on pentavalent 1, pentavalent 2, and pentavalent 3 timeliness at respective expanded programme immunisation due dates of 6 weeks, 10 weeks, and 14 weeks.

**Implications of all the available evidence**

Our research and existing evidence suggest that incentives were moderately useful in improving immunisation coverage and more effective at increasing immunisation timeliness. Cost-effectiveness analyses in relation to routine immunisation systems and outreach campaigns are needed. Additional studies that examine SMS reminders and monetary incentives in study populations with lower levels of immunisation coverage and timeliness are needed.

Another type of demand-generating intervention is the provision of conditional incentives for completing a desired behaviour. In LMICs, small monetary incentives have been applied to yield gains in the uptake of HIV testing and adult male circumcision.^20^, ^21^ In LMICs, mobile-money systems are frequently used instead of traditional banking systems and allow for the transfer of money through personal mobile phones.^22^ Aside from our previous pilot study,^16^ the use of mobile-money incentives to improve vaccination coverage has not been previously reported.

The Mobile Solutions for Immunization (M-SIMU) cluster-randomised controlled trial aimed to assess whether SMS reminders, either with or without mobile-money incentives, could improve the proportion of children fully immunised by their first birthday. Secondary outcomes included the effect of these interventions on vaccine-specific coverage and timeliness.

## Methods

### Study design and participants

The M-SIMU study was a four-arm, cluster-randomised controlled trial done within the Health and Demographic Surveillance System (HDSS) overseen by the Kenyan Medical Research Institute (KEMRI) and Centers for Disease Control and Prevention (CDC) in Siaya County, Nyanza Province. Clusters were villages, as defined by HDSS, and were randomly assigned and evenly allocated to one of four study groups: control, SMS reminders only (SMS only), SMS reminders plus a 75 Kenyan Shillings incentive (KES; SMS plus 75 KES, where 85 KES = USD$1 as of August, 2015) and, SMS reminders plus a 200 KES incentive (SMS plus 200 KES). A cluster-randomised approach was preferred to an individually randomised approach to minimise potential discord if neighbouring participants were randomised to different study groups. The conduct, analysis, and reporting of results were done in accordance with the Consolidated Standards of Reporting Trials (CONSORT) guidelines adapted for cluster-randomised trials.^23^

The M-SIMU study was done in rural, western Kenya, an area with high prevalence of HIV, tuberculosis, and malaria.^24^ Clusters (ie, villages) were included in the trial if they were located within Gem or Asembo districts and were within the HDSS boundaries. Clusters were excluded if they had ongoing special health programmes or immunisation activities that could bias the study outcomes.

The M-SIMU trial recruited HDSS village reporters to identify eligible caregivers and their infants. Village reporters used mobile phones to send birth notification text messages to the RapidSMS server. Birth notifications were relayed to field-based community interviewers who then screened caregivers of newborns for eligiblity into the study. Inclusion criteria for participation included being a caregiver of an infant aged 0–34 days and being a current resident of one of the randomised study villages. Exclusion criteria included being a caregiver planning to migrate from the study area in the next 6 months, if the infant received vaccination other than Bacillus Calmette–Guérin (BCG) or polio birth dose, or if the caregiver was not willing to vaccinate the child at an M-SIMU staffed clinic. Caregivers of infants aged 35 days and older were excluded because of the close temporal proximity to the first pentavalent visit scheduled at 6 weeks (ie, 42 days).^26^ M-SIMU health facility recorders were stationed at 24 clinics whose catchment area overlapped with all study villages.^24^ Mobile phone ownership was not an inclusion or exclusion criterion. Participants only needed to have access to a mobile phone, whereby access was defined by the caregiver. For those participants did not own a phone, the enrolment was paused until the participant confirmed with the owner of the shared phone that text messages and incentives, as applicable, could be sent to the mobile phone.

The protocol received ethical clearance from the Center Scientific Committee, Scientific Steering Committee (SSC), and the KEMRI-Nairobi Ethical Review Committee (ERC; SSC#2409). Johns Hopkins University Bloomberg School of Public Health and CDC deferred ethical clearance to KEMRI-ERC. A detailed description of the methods and protocol, including content of text message reminders and map of randomly assigned villages, has been reported.^25^ This trial is registered with ClinicalTrials.gov, number 01878435.

### Randomisation and masking

A baseline survey of vaccination coverage, phone ownership, and geographical and demographic characteristics was done on March 13 and April 29, 2013, to provide data for the randomisation.^27^ A constrained randomisation^28^ was done with GAUSS Mathematical and Statistical System by one of the study investigators, which randomly generated 1000 allocations that met the following criteria for balance across study arms: within a relative 10% for the means of full immunisation coverage, phone ownership, distance to the nearest clinic, and village population of children 12–23 months old; within a relative 25% within each district (Gem or Asembo) for the means of full immunisation coverage and phone ownership. The randomisation was stratified on district so that each study group contained exactly 30 villages from Gem and eight villages from Asembo.

The 1000 sequences were labelled with three-digit numbers, 000 to 999, each one assigning 38 villages to each of the four groupings (A–D). At a public randomisation ceremony on Sept 12, 2013, village chiefs determined the final randomisation outcome by picking numbered balls from a cloth sack to select one of these 1000 sequences, then picking labelled (study group) balls to assign the interventions to the chosen allocation.^24^

All caregivers and their infants (hereby referred to as infant-caregiver pairs) were allocated to the same study group as the randomised village in which they resided. If a caregiver moved during the follow-up period, the infant–caregiver pair retained their initial study group allocation. Due to the nature of the intervention and study design, study participants were not masked to their study group allocation. Field staff were not informed of a village's allocation, but this could be inferred from some enrolment and follow-up survey questions. Data cleaning was done by a statistician blinded to the allocation.

### Procedures

Participants provided written informed consent and were enrolled into the study by community interviewers after villages were randomly assigned. After obtaining consent, community interviewers sent an enrolment SMS to the RapidSMS server that contained the caregiver's phone number, the infant's birthdate, the preferred language to receive SMS reminders (English, Kiswahili, or Dholuo), and the infant's name.

All caregivers received a single text message at enrolment welcoming them to the study. For the three intervention groups, SMS reminders were sent three days and the day before scheduled immunisation visits at ages 6 weeks, 10 weeks, and 14 weeks for the three doses of pentavalent vaccine and age 9 months for measles vaccine using the free and open-source RapidSMS platform. Health facility recorders (HFR) were present at M-SIMU clinics to document immunisation. For immunised children, HFRs sent an SMS with the date of immunisations received and any change in caregiver's phone number to the RapidSMS server. For pentavalent 2 and pentavalent 3 vaccines, their respective due dates were recalculated to be 4 weeks from the texted pentavalent date (interval-appropriate schedule)^26^ and reminders were sent accordingly. Children who either went undocumented by the HFR or who did not receive a pentavalent vaccine had reminders sent at 6 weeks, 10 weeks, and 14 weeks.

In addition to receiving SMS reminders, caregivers were sent either 75 KES (group 3) or 200 KES (group 4) to their mobile phone for each timely dose of pentavalent and measles vaccine received, defined as vaccination within 2 weeks of the Expanded Programme on Immunisations (EPI) scheduled date (ie, pentavalent1 at 6 weeks, pentavalent2 and pentavalent3 4 weeks after the previous pentavalent dose, and measles at 9 months).^26^ Incentives were sent to participant's mobile phones using a mobile-money programme^22^ and through their preferred mobile network (eg, Safaricom, Airtel).

For children who were in the two incentive groups, the RapidSMS system log was downloaded daily and mobile-money delivered to caregivers whose children were timely vaccinated. Community interviewers did household follow-up visits when children reached 12 months of age to document the child's immunisation status with the maternal and child health (MCH) booklet. If the MCH booklet was not available, a verbal report of immunisation history was taken.

### Outcomes

The primary outcome was the proportion of fully immunised children by 12 months of age, defined as receiving BCG, three doses of polio vaccine, three doses of pentavalent vaccine, and measles vaccine. Polio birth dose was excluded from the primary outcomes definition because this vaccine is recommended to be received within the first 2 weeks of life. BCG was included because it is recommended up to 59 months of age and could be received at any of the pentavalent or measles vaccination visits.^26^ Vaccination coverage at 12 months of age and timely vaccination for pentavalent, polio, and measles vaccines were predetermined as secondary outcomes. Vaccination timeliness was defined as receiving vaccination within 2 weeks of the EPI due date for individual vaccines. A timely fully immunised child was defined as being fully immunised within 2 weeks of the measles EPI due date. Sensitivity analyses of pentavalent timeliness used an interval-appropriate schedule for calculating due dates of pentavalent2 and pentavalent3 vaccines, where the new pentavalent due date was calculated to be 4 weeks after the receipt of the previous pentavalent vaccine.

Data for primary and secondary outcomes came from written immunisation records found on the child's MCH booklet at 12-month follow-up visits. As available, MCH records were compared with prospectively collected immunisation records by HFRs. If there were discrepancies between MCH and prospectively-collected data, the health facility immunisation registries were consulted. If the registries did not resolve the discrepancy, MCH data were used. The MCH booklet was chosen as the primary source document because it was independent of the HFR-collected data. Infant–caregiver pairs were considered lost to follow-up if they outmigrated or the infant died before 12 months of age. Verbal reports of immunisation at 12-month household follow-up visits, in the absence of written documentation, were excluded from the analytic sample.

### Statistical analysis

Sample size calculations found that 152 villages were needed to detect a 15% absolute difference in the proportion of fully immunised children at 12 months of age between the control group and any given intervention group. A priori, we selected a 15% difference as a meaningful outcome as this represents an effect size that would motivate policy makers to adapt the interventions. The following assumptions were made for sample size calculations: a baseline coverage of fully immunised children of 70% at 12 months, village birth cohort with harmonic mean of 16 newborns, a between-cluster coefficient of variation (k) of 0·25, a 25% loss to follow-up, a type I error (α) of 0·05, and power (1-beta) of 0·80.

Primary analyses were done with modified intention-to-treat analyses at the participant level so that participants' outcomes were analysed regardless of the degree of exposure to study interventions. The term modified refers to the requirement of being able to determine the 12-month immunisation outcomes. Risk ratios for primary and secondary outcomes were calculated for the intervention groups compared with the control group using log-binomial regression^29^ and General Estimating Equations (GEE) to account for correlation within clusters. As a secondary analysis of the primary outcome, time-to-immunisation curves were constructed with the Kaplan–Meier method. To assess the heterogeneity of treatment effects by various risk factors at baseline, the log-binomial models were extended and interaction terms were tested. Subgroup analyses by phone ownership and time to clinic were prespecified. An additional six subgroups were analysed by study group to explore potential effect modification. Based on the 18 post-hoc subgroup interaction analyses, about one statistically significant test of interaction (p<0·05) would be expected on the basis of chance alone. Socioeconomic status was derived from multiple correspondence analysis of household possessions to produce quintile scores and then dichotomised into bottom 40% and upper 60%. Years of maternal education were collected as a continuous variable and dichotomised to align with the number of years needed to complete primary school in Kenya (8 years).

Per-protocol analyses of SMS reminders delivered were done for primary and secondary outcomes; per-protocol was defined as being sent the appropriate number of reminders per vaccine. Sensitivity analyses of vaccination coverage and timeliness were done with an interval-appropriate schedule of the pentavalent series. Analyses were done with STATA/SE (version 14.1; Stata Corp, College Station, TX, USA). An α of 0·05 was assumed for all statistical tests of significance.

### Role of the funding source

The funder of the study had no role in study design, data collection, data analysis, data interpretation, or writing of the report. The corresponding author had full access to all the data in the study and had final responsibility for the decision to submit for publication.

## Results

**Figure 1:**
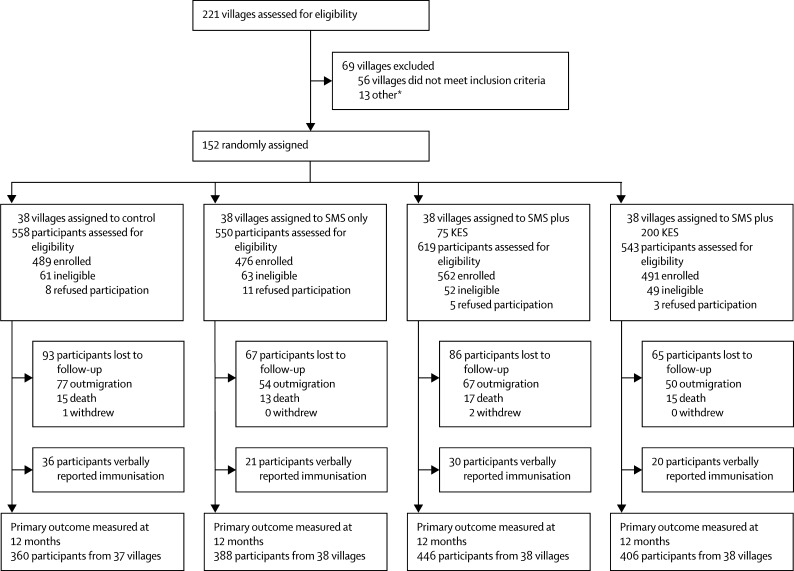
Trial profile SMS=short message service. KES=Kenyan Shilling. *Villages that immediately bordered villages that were excluded because of eligibility requirements.

Between Oct 14, 2013, and Oct 17, 2014, we enrolled 2018 caregivers and their infants (infant-caregiver pairs) from 152 villages (figure 1) to meet sample size requirements. The number of infant-caregiver pairs enrolled in control, SMS only, SMS plus 75 KES, and SMS plus 200 KES groups were 489 infant-caregiver pairs (38 villages), 476 infant-caregiver pairs (38 villages), 562 infant-caregiver pairs (38 villages), and 491 infant-caregiver pairs (38 villages), respectively. After accounting for loss to follow-up, which included outmigration and death, and excluding caregivers who verbally reported their child's immunisation history, the primary analytic sample contained 1600 infant–caregiver pairs who completed a 12-month follow-up survey and provided MCH booklet information for immunisation history; 360 (74%) infant–caregiver pairs from 37 villages (control arm), 388 (82%) pairs from 38 villages (SMS only), 446 (79%) pairs from 38 villages (SMS plus 75 KES), and 406 (83%) pairs from 38 villages (SMS plus 200 KES). Approximately 1600 (94%) of 1707 children who were alive and had not outmigrated had an MCH booklet present at 12-month follow-up. Sociodemographic characteristics of the analytic sample were similar across the four groups; notably, 49% of enrolled caregivers reported owning a mobile phone (table 1). Of the 810 (51%) of 1600 participants who shared a mobile phone, 456 (56%) of 810 used their husband's phone to receive text message reminders and incentives (appendix). Caregivers in the analytic sample were more likely to be older and have more children aged younger than 5 years old compared with those who were lost to follow-up or who did not have an MCH booklet (appendix). The coefficient of intercluster variation (*k)* for the primary outcome, a fully immunised child by 12 months of age, was calculated to be 0·089 in control group participants.

Data from the RapidSMS system's log showed that, overall, 4200 (85%) of 4960 vaccine doses (pentavalent 1–3 and measles) had two SMS reminders sent per protocol, and 4797 (97%) of 4960 vaccine doses had at least one SMS reminder sent (appendix). Mobile-money incentives were sent to caregivers for all timely vaccines given at M-SIMU clinics; 54% of incentives were delivered within 48 h of the immunisation date (data not shown).

**Figure 2:**
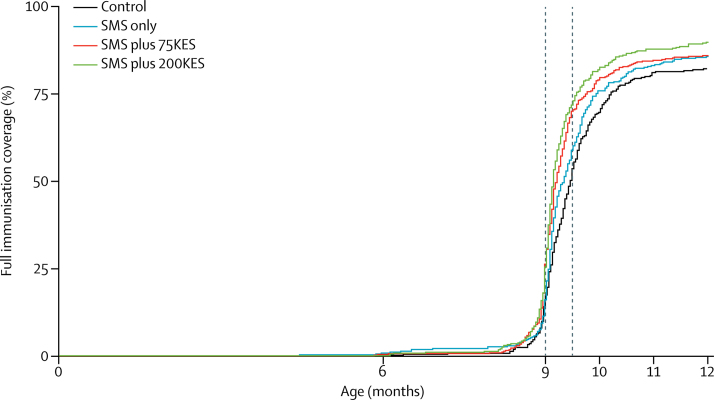
Effect of interventions on time to immunisation SMS=short message service. KES=Kenyan Shilling.

The proportions of children achieving the primary outcome, full immunisation by 12 months of age, were 82% (296 of 360) in control, 86% (332 of 388) in SMS only, 86% (383 of 446) in SMS plus 75 KES, and 90% (364 of 406) in SMS plus 200 KES groups (table 2). Children who were in villages randomly assigned to receive SMS reminders plus a 200 KES incentive were more likely to achieve full immunisation (risk ratio [RR] 1·09, 95% CI 1·02–1·16, p=0·014) than control group children. There were no significant differences in the primary outcome in either the SMS only or SMS plus 75 KES groups compared with the control arm. As a secondary outcome, children in the SMS plus 200 KES group were significantly more likely to receive measles vaccination by 12 months of age (1·07, 1·01–1·14, p=0·034) than control group children. There were no significant differences in secondary outcomes of vaccine-specific coverage estimates in SMS only and SMS plus 75 KES groups compared with the control group. Per-protocol analyses for delivering SMS reminders found similar estimates of vaccination coverage and associations with study groups as intention-to-treat analyses (appendix). As a secondary analysis, the median ages of achieving full immunisation with survival analysis were 288 days (IQR 277–311) in the control group, 284 (IQR 276–302) in the SMS only group, 280 (IQR 273–297) in the SMS plus 75 KES group, and 278 (IQR 273–291) in the SMS plus 200 KES group (figure 2). Bimodality in delayed immunisation was not observed (appendix)

The secondary outcome of achieving timely full immunisation, defined as being fully immunised within 2 weeks of the measles vaccine EPI due date, was significantly higher in all three intervention groups compared with the control group (table 3): SMS only (RR 1·18, 95% CI 1·01–1·39, p=0·045), SMS plus 75 KES (1·37, 1·18–1·59, p<0·0001), and SMS plus 200 KES (1·42, 1·23–1·65, p<0·0001). Exploratory analyses found that 41% (148 of 360), 48% (187 of 388), 60% (266 of 446), and 62% (252 of 406) of control, SMS only, SMS plus 75 KES, and SMS plus 200 KES children, respectively, received all pentavalent series and measles vaccines within 2 weeks of their respective EPI due dates of 6 weeks, 10 weeks, 14 weeks, and 9 months. Coverage was significantly higher in the SMS plus 75 KES (1·42, 1·19–1·71, p<0·0001) and SMS plus 200 KES (1·52, 1·27–1·81, p<0·0001) groups.

Risk ratios for timely measles vaccination were similar to timely fully immunised child estimates (table 3). Children in the SMS plus 200 KES group were more likely to receive pentavalent 3 vaccination within 2 weeks of the EPI due date compared with children in the control group (RR 1·12, 95% CI 1·03–1·22, p=0·0092), but were no longer significantly associated with timely pentavalent3 vaccination when applying an interval-appropriate schedule to the pentavalent series (appendix). We observed no effect of the interventions on timely vaccination for either pentavalent1 or pentavalent2 vaccination (table 3). Similar findings for vaccination timeliness were observed in per-protocol analyses, with the exception of pentavalent 2; which had significantly higher timeliness in study groups SMS plus 75 KES (1·06, 1·00–1·12, p=0·045) and SMS plus 200 KES (1·09, CI 1·03–1·15, p=0·002; appendix) than in the control group.

We found significant effects of SMS plus 200 KES in many of the disadvantaged populations, including those whose caregivers share a mobile phone, who are female children, who have caregivers in the bottom 40% bracket of socioeconomic status, who lived more than 30 min walking time to a clinic, and who had less than 8 years of education (table 4). We found significant stratum-specific risk ratios for maternal education across the three study groups compared with the control group, although the overall interaction was not significant (p=0·08, 3 degrees of freedom test). Children of caregivers with up to 7 years of education were more likely to be fully immunised than those with caregivers who had 8 or more years of education in the SMS group (RR 1·26, 95% CI 1·04–1·52, p=0·020), SMS plus 75 KES group (1·28, 1·06–1·53, p=0·012), and SMS plus 200 KES group (1·29, 1·07–1·55, p=0·039) compared with children in the control group. We observed similar findings of the intervention's effect on traditionally marginalised populations for timely measles and pentavalent3 vaccination (appendix). Subgroup analyses for pentavalent1 and pentavalent2 vaccinations were not done because of high levels of timeliness in the control group.

The vast majority of caregivers reported receiving at least one SMS reminder or incentive during the study and that these interventions were influential in their decision to vaccinate their child (table 5). The three most common uses of incentives were to pay for housing expenditures (342 of [46%] 747), purchase food (247 [33%] of 747), and for transportation (67 [9%] of 747). All caregivers, except one, said they would retain their enthusiasm for vaccination for a future child even if the incentives were not given. We observed no indirect benefit of interventions on health outcomes or non-vaccine health seeking behaviours (appendix). In analyses that adjusted for demographic differences between the analytic sample and participants who were lost to follow-up, children in the SMS plus 200 KES group were less likely to outmigrate than those in the control group.

## Discussion

This study shows that in a rural sub-Saharan African setting with high baseline immunisation coverage, SMS reminders plus monetary incentives were modestly effective at improving the proportion of children fully vaccinated by 12 months of age and SMS reminders, with or without incentives, yielded significant gains in timeliness of measles vaccination. Importantly, the incentives seemed to have an equitable effect, significantly improving timely vaccination across measured sociodemographic characteristics. Lower-income families must balance the direct and opportunity costs of seeking health care with the perceived benefits of curative and preventive services, where individuals might place greater emphasis on benefits that are immediate, such as treatment, compared with those that are delayed, like preventive services and immunisations.^30^ In our study and others,^31^, ^32^ the use of small monetary incentives might have served as a nudge for caregivers to seek timely immunisation services for their children. This study adds to the growing body of scientific literature that shows strong effects of demand-side interventions for immunisation coverage in LMICs.^33^

Notably, we were able to observe significant effects of the mobile phone-delivered interventions in a study population comprised equally of those who reported owning or sharing a mobile phone. By enrolling caregivers who shared a mobile phone, it was implicit that caregivers would need to discuss with the phone owner that SMS messages and incentives would need to be relayed from phone owner to intended recipient. More than 90% of enrolled caregivers reported receiving at least one SMS or incentive, as applicable. When combined with our subgroup analyses, this result suggests that the interventions were successfully relayed. The exact nature of this transfer, and whether there were any informal transactional costs in those who received incentives, is not well understood, but future focus group discussions might provide further insight.

As the use of economic incentives becomes more widespread, there have been considerable discussions regarding the ethics of this approach.^34^ The incentive amounts in this study were not coercive; they were less than one day's working wage^21^ and were targeted to a routine care-seeking practice and not paired to risky or dangerous behaviour. Incentives were effective in many of the marginalised subpopulations and therefore did not seem to exacerbate any existing inequities. Future studies need to address whether their application created a dependency. If incentives can improve immunisation coverage and timeliness, subsequently higher coverage levels would induce herd immunity for some of the vaccine preventable diseases and confer health and economic benefits to people who do not receive incentives.

A common critique of incentives focuses on sustainability and scalability. To address these concerns, first, replication studies, including urban settings and areas with low immunisation coverage, are needed before recommending incentives as a way for improving immunisation rates. Second, incentives must be cost-effective. Aside from the economic savings associated with treatment cost and productivity loss,^35^ the use of incentives, at least in this study area, might be cost-effective or even cost-neutral if they significantly strengthen the routine immunisation services, thereby reducing the need for supplemental and out-reach immunisation activities. Costing analyses are underway at present. Lastly, the application of incentives could be selectively targeted to districts with poor immunisation rates or for particular vaccines that exhibit low population coverage levels, such as measles or human papillomavirus. A targeted approach of delivering incentives to subpopulations within Kenya is not novel.^36^

This study has several strengths. First, selection bias was minimised by enrolling infants at home shortly after birth, rather than enrolling from a health facility. Enrolment of infants before their first vaccination ensures that the study sample was representative of its population by including infants who might not go for any vaccines. Second, this trial was designed and implemented to closely mimic an effectiveness study and with scalability in mind by enrolling caregivers independent of mobile phone ownership, a study design unlike some m-health intervention trials that provide a mobile phone^7^ or require one for enrolment.^8^, ^14^, ^37^ We do not believe the high coverage estimates observed in the control group were due to study participation (ie, Hawthorne effect) because the coverage was similar to that in a baseline survey done before the trial.^27^ Moreover, we sought to minimally interfere with participants' care-seeking behaviours because household visits were only done at enrolment and 12-month follow-up. Lastly, our per-protocol analyses of SMS reminder delivery and sensitivity analyses of pentavalent interval-appropriate schedules largely mirrored our intention-to-treat and MCH booklet data source analyses.

The absence of a significant association between participants in the SMS only group and the primary outcome of immunisation coverage at 12 months contradicts positive findings from the majority of SMS reminder randomised controlled trials done in sub-Saharan Africa and Kenya.^6^, ^7^, ^8^, ^9^, ^10^, ^11^ However, we found SMS reminders significantly improved timely measles immunisation, a secondary outcome. The success of SMS reminders to elicit a behaviour is multifactorial; the content of the message, the type of behaviour being reminded, indirect and direct costs incurred, literacy level, and other contextual factors all being potential explanatory factors. SMS reminders were probably not effective at improving full immunisation and vaccine-specific coverages in this study because of high baseline coverage levels and because SMS reminders might not have addressed the demand-side deficiencies in this study area. Similarly, investigators have reported that two-way messages are more effective than one-way messages, as used in M-SIMU, for medication adherence.^38^ Future studies using SMS reminders in areas of low vaccination coverage are needed.

This study has several limitations. First, our analytic sample consisted of 79% of enrolled caregiver–infant pairs. We anticipated a loss to follow-up given the study area's trends in outmigration^25^ and adjusted sample size accordingly. There were some differences in sociodemographic characteristics between the analytic sample and those who were excluded from the analysis. Excluded caregivers were more likely to be less than 25 years old, have one child younger than 5 years old in the household, and reside in Gem. In this area, there is a cultural practice of caregivers migrating to their home village to give birth, which might be more common in younger mothers who are having their first child. Second, although we report high levels of successfully delivering SMS reminders and incentives, we do not have complete information on whether SMS reminders were received and read or if reminders and incentives were relayed to enrolled caregivers in those who shared a mobile phone. Lastly, although BCG vaccine was not paired with the intervention, we included it in our primary outcome for concordance with global definitions of fully immunised child. The effect of BCG's inclusion on our estimates of full immunisation was minimal: of the nine children who did not receive BCG, five children would have been classified as not achieving full immunisation for missing other vaccinations.

This rural study area has high baseline immunisation levels, moderate levels of mobile phone ownership,^27^ and a widespread mobile-money network.^22^ Replicating this trial in settings where mobile phone ownership is lower and mobile-money systems are absent or less frequently used might not be possible. Nevertheless, components of the intervention can be disaggregated into individual modules (SMS reminders, incentives, and mobile-money) and can be applied based on a country's available technology. For example, instead of transferring cash via mobile-money, incentives can be given by providing mobile phone airtime or even cash vouchers.^32^ Mobile-money was used in this trial because of its widespread acceptance, its logistical ease of delivery, and the avoidance of having cash at a health facility where it might present security risks and either be lost or stolen.

In conclusion, the provision of SMS reminders coupled with small monetary incentives led to significant, though modest improvements in fully immunised child coverage and larger gains in immunisation timeliness. We found that the interventions were equally effective across all subgroups. In other resource-constrained settings where immunisation coverage is low, it is likely that SMS reminders, with or without incentives, could raise immunisation timeliness, but additional research is needed.

## Figures and Tables

**Table 1:** Demographics and baseline characteristics of study participants from Gem and Asembo districts (Kenya), 2013–15

		**Control (n=360)**	**SMS only (n=388)**	**SMS plus 75 KES (n=446)**	**SMS plus 200 KES (n=406)**	**Total (n=1600)**
Average cluster size		10 (6)	10 (5)	12 (6)	11 (6)	11 (6)
Mobile phone access						
	Shares phone	178 (49%)	183 (47%)	236 (53%)	213 (52%)	810 (51%)
	Owns phone	182 (51%)	205 (53%)	210 (47%)	193 (48%)	790 (49%)
Mobile network						
	Safaricom	343 (95%)	378 (97%)	440 (99%)	396 (98%)	1557 (97%)
	Other	17 (5%)	10 (3%)	6 (1%)	10 (1%)	43 (3%)
Infant's sex						
	Female	186 (52%)	179 (46%)	228 (51%)	207 (51%)	800 (50%)
	Male	174 (48%)	209 (54%)	218 (49%)	199 (49%)	800 (50%)
Infant age at enrolment (days)		14 (8)	14 (8)	14 (8)	14 (8)	14 (8)
Socioeconomic status						
	Bottom 40%	132 (37%)	144 (37%)	181 (41%)	172 (42%)	629 (39%)
	Upper 60%	228 (63%)	244 (63%)	265 (59%)	234 (58%)	971 (61%)
Time to clinic						
	≤30 min	202 (56%)	225 (58%)	293 (66%)	255 (63%)	975 (61%)
	>30 min	158 (44%)	163 (42%)	153 (34%)	151 (37%)	625 (39%)
Maternal education						
	≤7 years	83 (23%)	97 (25%)	124 (28%)	107 (26%)	411 (26%)
	>7 years	277 (77%)	291 (75%)	322 (72%)	299 (74%)	1189 (74%)
Maternal age*						
	≤25 years	174 (49%)	203 (53%)	221 (50%)	227 (56%)	825 (52%)
	>25 years	184 (51%)	183 (47%)	223 (50%)	179 (44%)	769 (48%)
Number of children younger than 5 years in house						
	≤1	122 (34%)	133 (34%)	146 (33%)	157 (39%)	558 (35%)
	>1	238 (66%)	255 (66%)	300 (67%)	249 (61%)	1042 (65%)
Region						
	Asembo	75 (21%)	87 (22%)	92 (21%)	81 (20%)	335 (21%)
	Gem	285 (79%)	301 (78%)	354 (79%)	325 (80%)	1265 (79%)
Place of last delivery						
	At home	83 (23%)	110 (28%)	139 (31%)	113 (28%)	445 (28%)
	Health Facility	277 (77%)	278 (72%)	307 (69%)	293 (72%)	1155 (72%)

Data are mean (SD) or n (%). SMS=short message service. KES=Kenyan Shilling.

* Six missing values.

**Table 2:** Effects of interventions on primary outcome of full vaccination coverage at 12 months of age in study participants from Gem and Asembo districts, Kenya, 2013–15

	**Control (n=360)**	SMS only (n=388)*	**p value**†	SMS plus 75 KES (n=446)*	**p value**†	SMS plus 200 KES (n=406)*	**p value**†
**Primary outcome at 12 months**							
Fully immunised child‡	296 (82%)	332 (86%); 1·04 (0·97–1·12)	0·29	383 (86%); 1·04 (0·96–1·11)	0·33	364 (90%); 1·09 (1·02–1·16)	0·014
**Vaccines in primary outcome**							
BCG§	360 (100%)	382 (98%); 0·99 (0·82–1·18)	0·88	444 (100%); 1·00 (0·83–1·19)	0·96	405 (100%); 1·00 (0·83–1·19)	0·97
Pentavalent1§	359 (100%)	387 (100%); 1·00 (0·87–1·15)	0·99	444 (100%); 1·00 (0·87–1·014)	0·98	406 (100%); 1·00 (0·87–1·15)	0·97
Pentavalent2	356 (99%)	383 (99%); 1·00 (0·98–1·01)	0·77	442 (99%); 1·00 (0·99–1·02)	0·85	404 (100%); 1·01 (0·99–1·02)	0·42
Pentavalent3	353 (98%)	375 (97%); 0·98 (0·96–1·01)	0·20	439 (98%); 1·00 (0·98–1·02)	0·82	401 (99%); 1·01 (0·99–1·02)	0·58
Polio1§	359 (100%)	386 (99%); 1·00 (0·87–1·14)	0·97	444 (100%); 1·00 (0·87–1·14)	0·98	406 (100%); 1·00 (0·88–1·15)	0·97
Polio2	355 (99%)	383 (99%); 1·00 (0·98–1·02)	0·91	442 (99%); 1·00 (0·99–1·02)	0·76	404 (100%); 1·01 (0·99–1·02)	0·40
Polio3	349 (97%)	372 (96%); 0·98 (0·95–1·01)	0·29	436 (98%); 1·00 (0·98–1·03)	0·85	401 (99%); 1·01 (0·99–1·04)	0·30
Measles	302 (84%)	338 (87%); 1·04 (0·97–1·11)	0·28	388 (87%); 1·03 (0·97–1·10)	0·36	365 (90%); 1·07 (1·01–1·14)	0·034

Data are n (%) and RR (95% CI) for 1600 children with immunisation data recorded on maternal and child health booklet at 12 months. SMS=short message service. KES=Kenyan Shilling.

* Risk ratios and 95% CIs were calculated using General Estimating Equations with an exchangeable correlation matrix to account for correlation within clusters.

† Compared with the control group.

‡ k=0·089 in the control group; k=0·069 in the SMS only group; k=0·073 in the SMS plus 75 KES group; k=0·053 in the SMS plus 200 KES group.

§ Poisson error distribution used because of model convergence issues.

**Table 3:** Effect of interventions on secondary outcome of vaccination timeliness in study participants from Gem and Asembo districts, Kenya, 2013–15

		**Control (n=360)**	SMS only (n=388)*	**p value**†	SMS plus 75 KES (n=446)*	**p value**†	SMS plus 200 KES (n=406)*	**p value**†
Fully immunised child‡		181 (50%)	228 (59%); 1·18 (1·00–1·39)	0·045	312 (70%); 1·37 (1·18–1·59)	<0·0001	291 (72%); 1·42 (1·23–1·65)	<0·0001
Vaccines in fully immunised children§								
	Pentavalent1	328 (91%)	347 (89%); 0·98 (0·94–1·03)	0·47	412 (92%); 1·01 (0·97–1·06)	0·55	377 (93%); 1·02 (0·98–1·06)	0·40
	Pentavalent2	303 (84%)	320 (82%); 0·98 (0·92–1·05)	0·54	387 (87%); 1·03 (0·97–1·09)	0·31	359 (88%); 1·05 (0·99–1·11)	0·093
	Pentavalent3	267 (74%)	288 (74%); 1·01 (0·91–1·11)	0·90	354 (79%); 1·07 (0·98–1·17)	0·16	337 (83%); 1·12 (1·03–1·22)	0·0092
	Measles	183 (51%)	231 (60%); 1·18 (1·01–1·38)	0·038	316 (71%); 1·37 (1·19–1·59)	<0·0001	292 (72%); 1·42 (1·23–1·63)	<0·0001
Receiving all timely vaccines§		148 (41%)	187 (48%); 1·20 (0·98–1·46)	0·075	266 (60%); 1·42 (1·19–1·71)	0·0001	252 (62%); 1·52 (1·27–1·81)	<0·0001

Data are n (%) and risk ratio (95% CI) for 1600 children with immunisation data recorded on maternal and child health booklet at 12 months. SMS=short message service. KES=Kenyan Shilling.

* Risk ratios and 95% CI were adjusted to account for correlation within clusters.

† As compared to control arm.

‡ Timeliness defined as receiving vaccination within 2 weeks of EPI due date and for fully immunised child, 2 weeks within the measles due date.

§ Children who received pentavalent 1, pentavalent 2, pentavalent 3, and measles vaccines within 2 weeks of their respective EPI due date.

**Table 4:** Subgroup analyses of full immunisation coverage at 12 months of age in study participants from Gem and Asembo districts, Kenya, 2013–15

	**Control (n=360)**	**SMS (n=388)**	**Stratum-specific RR**	p value*	**SMS plus 75 KES (n=446)**	**Stratum-specific RR**	p value*	**SMS plus 200 KES (n=406)**	**Stratum-specific RR**	p value*
**Phone access**										
Owns	156/182 (86%)	181/205 (88%)	1·03 (0·95–1·12)	0·79	183/210 (87%)	1·01 (0·92–1·10)	0·35	179/193 (93%)	1·08 (1·00–1·16)	0·72
Shares	140/178 (79%)	151/183 (83%)	1·05 (0·94–1·17)	··	200/236 (85%)	1·07(0·96–1·18)	··	185/213 (87%)	1·10 (1·00–1·22)	··
**Infant sex**										
Male	146/174 (84%)	177/209 (85%)	1·01 (0·93–1·10)	0·34	188/218 (86%)	1·03 (0·95–1·12)	0·61	181/199 (91%)	1·08 (1·00–1·17)	0·85
Female	150/186 (81%)	155/179 (87%)	1·07 (0·98–1·18)	··	195/228 (86%)	1·06 (0·97–1·16)	··	183/207 (88%)	1·10 (1·01–1·19)	··
**Socioeconomic status**										
Bottom 40%	101/132 (77%)	112/144 (78%)	1·02 (0·89–1·16)	0·62	153/181 (85%)	1·10 (0·99–1·24)	0·21	152/172 (88%)	1·15 (1·04–1·29)	0·19
Top 60%	195/228 (86%)	220/244 (90%)	1·05 (0·99–1·13)	··	230/265 (87%)	1·01 (0·94–1·09)	··	212/234 (91%)	1·06 (0·99–1·13)	··
**Time to clinic**										
≤30 min	167/202 (83%)	193/225 (86%)	1·04 (0·96–1·13)	0·92	255/293 (87%)	1·05 (0·97–1·14)	0·68	226/255 (89%)	1·07 (0·99–1·16)	0·47
>30 min	129/158 (82%)	139/163 (85%)	1·04 (0·95–1·15)	··	128/153 (84%)	1·02 (0·93–1·13)	··	138/151 (91%)	1·12 (1·02–1·22)	··
**Maternal education**										
≤7 years	53/83 (64%)	78/97 (80%)	1·26 (1·04–1·52)	0·020	101/124 (81%)	1·28 (1·06–1·53)	0·012	88/107 (82%)	1·29 (1·07–1·55)	0·039
>7 years	243/277 (88%)	254/291 (87%)	0·99 (0·94–1·06)	··	282/322 (88%)	1·00 (0·94–1·06)	··	276/299 (92%)	1·05 (1·00–1·11)	··
**Maternal age**†										
≤25 years	147/174 (84%)	179/203 (88%)	1·04 (0·96–1·13)	0·87	201/221 (91%)	1·08 (1·00–1·16)	0·34	206/227 (91%)	1·07 (1·00–1·16)	0·72
>25 years	148/184 (80%)	152/183 (83%)	1·03 (0·94–1·14)	··	182/223 (82%)	1·01 (0·92–1·12)	··	158/179 (88%)	1·10 (1·00–1·20)	··
**Children younger than 5 years in the house**										
≤1	105/122 (86%)	118/133 (89%)	1·03 (0·94–1·13)	0·82	132/146 (90%)	1·05 (0·96–1·15)	0·90	146/157 (93%)	1·08 (0·99–1·17)	0·87
1	191/238 (80%)	214/255 (84%)	1·05 (0·96–1·14)	··	251/300 (84%)	1·04 (0·96–1·13)	··	218/249 (88%)	1·09 (1·01–1·18)	··
**Region**										
Gem	228/285 (80%)	254/301 (84%)	1·05 (0·97–1·15)	0·45	303/354 (86%)	1·06 (0·98–1·15)	0·21	289/325 (89%)	1·11 (1·02–1·20)	0·28
Asembo	68/75 (91%)	78/87 (90%)	1·00 (0·89–1·12)	··	80/92 (87%)	0·96 (0·85–1·09)	··	75/81 (93%)	1·03 (0·93–1·14)	··

Data are n/N (%) and RR (95% CI) for 1600 children with immunisation data recorded on maternal and child health booklet at 12 months. SMS=short message service. KES=Kenyan Shilling. RR=risk ratios.

* p values obtained from an interaction term between intervention groups and risk factor.

† Six missing values.

**Table 5:** Opinions of SMS reminders and mobile-money incentives from study participants from Gem and Asembo districts, Kenya, 2013–15

	**SMS only**	**SMS plus 75 KES**	**SMS plus 200 KES**
**Received any SMS reminder during study**			
Yes	354/382 (93%)	419/446 (94%)	394/405 (97%)
Caregiver's opinion on number of SMS reminders per vaccine*			
Just right	274/281 (98%)	310/316 (98%)	302/307 (98%)
Too few	3/281 (1%)	6/316 (2%)	3/307 (1%)
Too many	4/281 (1%)	0/316 (<1%)	2/307 (1%)
**SMS influenced decision to vaccinate child**			
Yes	296/354 (84%)	355/419 (85%)	332/394 (84%)
**Received any incentive during study**			
Yes	··	387/446 (87%)	360/406 (89%)
**Incentive influenced decision to vaccinate child**			
Yes	··	304/387 (79%)	260/360 (72%)
**Intervention most influential for decision to vaccinate child**			
SMS	··	253/387 (65%)	236/360 (66%)
Incentive	··	30/387 (8%)	31/360 (9%)
Neither	··	12/387 (3%)	12/360 (3%)
SMS and incentive equally	··	92/387 (24%)	81/360 (23%)
**Likelihood of vaccinating future child without an incentive**			
Less likely	··	0/387	1/360 (<1%)
The same	··	114/387 (29%)	151/360 (42%)
More likely	··	273/387 (71%)	208/360 (58%)
**Incentive used for**†			
Housing expenses	··	164/387 (42%)	178/360 (49%)
Food	··	116/387 (30%)	131/360 (36%)
Transportation	··	52/387 (13%)	15/360 (4%)
Clothes for child	··	8/377 (2%)	31/360 (9%)
Mobile phone airtime	··	34/387 (9%)	1/360 (<1%)
Not used by mother	··	4/387 (1%)	1/360 (<1%)
Didn't cash out incentive	··	4/387 (1%)	1/360 (<1%)
Other	··	13/387 (3%)	14/360 (4%)

Data are n/N (%) and come from a household survey done at child's age of 12 months. SMS=short message service. KES=Kenyan Shilling.

* Data missing for 262 caregivers who reported sharing a mobile phone and not being able to provide an opinion.

† Percentages do not sum to 100% because caregivers could select more than one option.
