# Supplementary material for: Mobile phone-delivered reminders and incentives to improve childhood immunisation coverage and timeliness in Kenya (M-SIMU): a cluster randomised controlled trial
Source: Lancet Glob Health. 2017 Mar 11;5(4):e428–38. doi: 10.1016/S2214-109X(17)30072-4 (PMC5348605; doi:10.1016/S2214-109X(17)30072-4)
Supplement: Supplementary appendix [file mmc1.pdf]

THE LANCET
Global Health

Supplementary appendix

This appendix formed part of the original submission and has been peer reviewed. We post it as supplied by the authors.

Supplement to: Gibson DG, Ochieng B, Kagucia EW, et al. Mobile phone-delivered reminders and incentives to improve childhood immunisation coverage and timeliness in Kenya (M-SIMU): a cluster randomised controlled trial. *Lancet Glob Health* 2017; **5:** e428–38.

Appendix Table S1. Detailed distribution of mobile phone ownership and access at enrollment

Mobile Phone Access Point	Control (n=360)	SMS Only (n=388)	SMS+75KES (n=446)*	SMS+200KES (n=406)*	Total (n=1600)†	Shared Phone (n=810)†
Owns phone	182 (51%)	205 (53%)	210 (47%)	193 (48%)	790 (49%)	
Spouse	106 (29%)	107 (28%)	124 (28%)	119 (29%)	456 (29%)	456 (56%)
Someone else in household	27 (8%)	25 (6%)	44 (10%)	31 (8%)	127 (8%)	127 (16%)
Someone else in compound	26 (7%)	22 (6%)	29 (7%)	26 (6%)	103 (6%)	103 (13%)
Neighbor	12 (3%)	24 (6%)	33 (7%)	30 (7%)	99 (6%)	99 (12%)
Other	7 (2%)	5 (1%)	5 (1%)	6 (1%)	23 (1%)	23 (2%)

Data are n (%). SMS=short message service. KES=Kenyan Shilling. *1 missing value. † 2 missing values

Table S2. Demographics of analytic vs non-analytic sample

	Analytic sample (n=1600)	Verbal report (n=107)	LFU (n=311)	Total Excluded (n=418)	p value*
**Mobile phone access**					
Shares phone	810 (51%)	63 (59%)	167 (54%)	230 (55%)	0·11
Owns phone	790 (49%)	44 (41%)	144 (46%)	188 (45%)	
**Infant's gender**					
Female	800 (50%)	60 (56%)	159 (51%)	219 (52%)	0·38
Male	800 (50%)	47 (44%)	152 (49%)	199 (48%)	
**Socioeconomic status**					
Bottom 40%	629 (39%)	35 (33%)	144 (46%)	179 (43%)	0·19
Upper 60%	971 (61%)	72 (67%)	167 (54%)	239 (57%)	
**Time to clinic**					
≤ 30 minutes	975 (61%)	67 (63%)	199 (64%)	266 (64%)	0·31
> 30 minutes	625 (39%)	40 (37%)	112 (36%)	152 (36%)	
**Maternal Education**					
≤ 7 years	411 (26%)	22 (21%)	76 (24%)	98 (23%)	0·35
> 7 years	1189 (74%)	85 (79%)	235 (76%)	320 (77%)	
**Maternal Age†**					
≤ 25 years	825 (52%)	72 (67%)	228 (73%)	300 (72%)	0·0001
>25 years	769 (48%)	35 (33%)	83 (27%)	118 (28%)	
**# Child under 5 in house**					
≤1 child	558 (35%)	35 (33%)	145 (47%)	180 (43%)	0·002
>1 child	1042 (65%)	72 (67%)	166 (53%)	238 (57%)	
**Region**					
Asembo	335 (21%)	22 (21%)	46 (15%)	68 (16%)	0·033
Gem	1265 (79%)	85 (79%)	265 (85%)	350 (84%)	
**Place of last delivery**					
At home	445 (28%)	33 (31%)	82 (26%)	115 (28%)	0·90
Health Facility	1155 (72%)	74 (69%)	229 (74%)	303 (72%)	

Data are mean (SD) or n (%). Percentages do not add up to 100% in some cases due to rounding. LFU= loss to follow up. SMS=short-message service. LFU includes deceased children and caregivers who out-migrated from the study area. *As compared to analytic sample. †6 missing values in analytic sample.

Table S3. SMS reminders sent per protocol by study arm

	SMS (n=388)	SMS+75KES (n=446)	SMS+200KES (n=406)	Total (n=1240)
**Per Protocol***				
Pentavalent1	306 (78·9%)	367 (82·3%)	341 (84·0%)	1014 (81·8%)
Pentavalent2	284 (73·2%)	341 (76·5%)	317 (78·1%)	942 (76·0%)
Pentavalent3	309 (79·6%)	366 (82·0%)	338 (83·3%)	1013 (81·7%)
Measles	386 (99·5%)	442 (99·1%)	403 (99·3%)	1231 (99·3%)
Total	1285/1552 (82·8%)	1516/1784 (85·0%)	1399/1624 (86·1%)	4200/4960 (84·7%)
**Sent any SMS†**				
Pentavalent1	362 (93·3%)	415 (93·1%)	380 (93·6%)	1157 (93·3%)
Pentavalent2	366 (94·3%)	423 (94·8%)	388 (95·6%)	1177 (94·9%)
Pentavalent3	381 (98·2%)	442 (98·9%)	401 (98·8%)	1223 (98·6%)
Measles	387 (99·7%)	446 (100%)	406 (100%)	1239 (99·9%)
Total	1496/1552 (96·4%)	1726/1784 (96·7%)	1575/1624 (96·9%)	4797/4960 (96·7%)
**Sent no SMS‡**				
Pentavalent1	26 (6·7%)	31 (7·0%)	26 (6·4%)	83 (6·7%)
Pentavalent2	22 (5·7%)	23 (5·2%)	18 (4·4%)	63 (5·1%)
Pentavalent3	7 (1·8%)	5 (1·1%)	5 (1·2%)	17 (1·4%)
Measles	1 (0·3%)	0 (0%)	0 (0%)	1 (0·1%)
Total	56/1552 (3·6%)	59/1784 (3·3%)	49/1624 (3·0%)	164/4960 (3·3%)

Data are n (%) for 1240 children with immunisation data recorded on maternal and child health booklet at 12 months and in the intervention arms. SMS=short message service. KES=Kenyan Shilling. *Per protocol is defined as being sent the appropriate number of SMS reminders per vaccine. †Sent any SMS is defined as a caregiver who was sent reminders per protocol or sent at least one SMS. ‡Sent no SMS is defined as caregivers who were not sent a single SMS reminder for that vaccine.

Table S4. Per protocol analysis of SMS reminders on vaccination coverage at 12 months of age

Vaccine	Control (n=360)	SMS (n=388)*	p value†	SMS+75KES (n=446)*	p value†	SMS+200KES (n=406)*	p value†
**Per protocol‡**							
Penta1	359/360 (99·7%)	305/306 (99·7%)<br>1·00 (0·99–1·01)	0·89	365/367 (99·5%)<br>1·00 (0·99–1·01)	0·57	341/341 (100·0%)<br>N.A.†	N.A.‡
Penta2	356/360 (98·9%)	281/284 (98·9%)<br>1·00 (0·98–1·02)	0·93	337/341 (98·8%)<br>1·00 (0·98–1·01)	0·82	317/317 (100·0%)<br>N.A.†	N.A.‡
Penta3	353/360 (98·1%)	300/309 (97·1%)<br>0·99 (0·96–1·01)	0·38	359/366 (98·1%)<br>1·00 (0·98–1·02)	0·94	336/338 (99·4%)<br>1·01 (1·00–1·03)	0·15
Measles	302/360 (83·9%)	336/386 (87·1%)<br>1·04 (0·97–1·11)	0·28	384/442 (86·9%)<br>1·03 (0·96–1·10)	0·37	363/403 (90·1%)<br>1·07 (1·01–1·14)	0·028
**Sent any SMS§**							
Penta1	359/360 (99·7%)	361/362 (99·7%)<br>1·00 (0·99–1·01)	0·98	413/415 (99·5%)<br>1·00 (0·99–1·01)	0·63	379/379 (100·0%)<br>N.A.†	N.A.‡
Penta2	356/360 (98·9%)	361/366 (98·6%)<br>1·00 (0·98–1·02)	0·71	419/423 (99·1%)<br>1·00 (0·99–1·02)	0·90	386/388 (99·5%)<br>1·01 (0·99–1·02)	0·45
Penta3	353/360 (98·1%)	368/381 (96·6%)<br>0·98 (0·96–1·01)	0·19	434/441 (98·4%)<br>1·00 (0·98–1·02)	0·83	396/401 (98·8%)<br>1·01 (0·99–1·02)	0·59
Measles	302/360 (83·9%)	337/387 (87·1%)<br>1·04 (0·97–1·11)	0·28	388/446 (87·0%)<br>1·03 (0·97–1·10)	0·36	365/406 (89·9%)<br>1·07 (1·01–1·14)	0·034

Data are n (%) and RR (95% CI) for children with immunisation data recorded on maternal and child health booklet at 12 months. SMS=short message service. KES=Kenyan Shilling. *Risk Ratios and 95% CI were adjusted to account for correlation within clusters †As compared to control arm. ‡Study arm perfectly predicts outcome. ‡Per protocol SMS reminders defined as being sent the appropriate number of reminders per vaccine. §Sent any SMS is defined as a caregiver who was sent reminders per protocol or sent at least one SMS.

Table S5. Distribution in delays (days) for FIC and vaccines within FIC

	Recommended age	Control	SMS Only	SMS + 75KES	SMS +200KES
**Primary Outcome**					
FIC*	9 months (274 d)	10 (2, 20)<br>12·5 (19·2)	6 (5, 19)<br>9·4 (23·6)	4 (-1, 12)<br>6·7 (18·0)	3 (-1, 11)<br>6·4 (19·4)
**Days delayed**					
BCG	Birth (0 d)	11 (4, 19)<br>16·3 (21·8)	11 (5, 17)<br>16·5 (29·1)	12 (5, 20)<br>16·9 (26·0)	10 (4, 18)<br>13·7 (17·1)
Pentavalent1	6 weeks (42 d)	1 (0, 5)<br>4·7 (11·3)	2 (0, 6)<br>5·8 (18·2)	1 (0, 4)<br>4·0 (16·9)	1 (0, 5)<br>3.8 (9·6)
Pentavalent2	10 weeks (70 d)	4 (1, 10)<br>8·5 (18·5)	5 (1, 10)<br>9·1 (21·1)	3 (0, 8)<br>6·4 (16·7)	3 (0, 7)<br>6·9 (15·4)
Pentavalent3	14 weeks (98 d)	6 (2, 14)<br>11·7 (19·9)	7 (2, 14)<br>12·7 (23·4)	5 (1, 12)<br>9·8 (17·7)	4 (1, 11)<br>8·7 (18·3)
Measles	9 months (274 d)	10 (2, 20)<br>12·7 (19·2)	6 (0, 19)<br>9·6 (23·9)	4 (-1, 12)<br>6·7 (18·0)	3 (-1, 11)<br>6·3 (19·3)

Data are median (IQR) and mean (SD) for vaccination delays (days) by study arm. Delays are counted from the recommended age. Children who did not receive vaccination are excluded from analyses. SMS=short message service. KES=Kenyan Shilling. FIC=fully immunised child. *FIC defined as a child who received BCG, three doses of pentavalent vaccine, three doses of polio vaccine, and measles vaccine by 12 months of age.

Figure S1 Histogram for delays (days) of pentavalent series and measles vaccine by study arms.

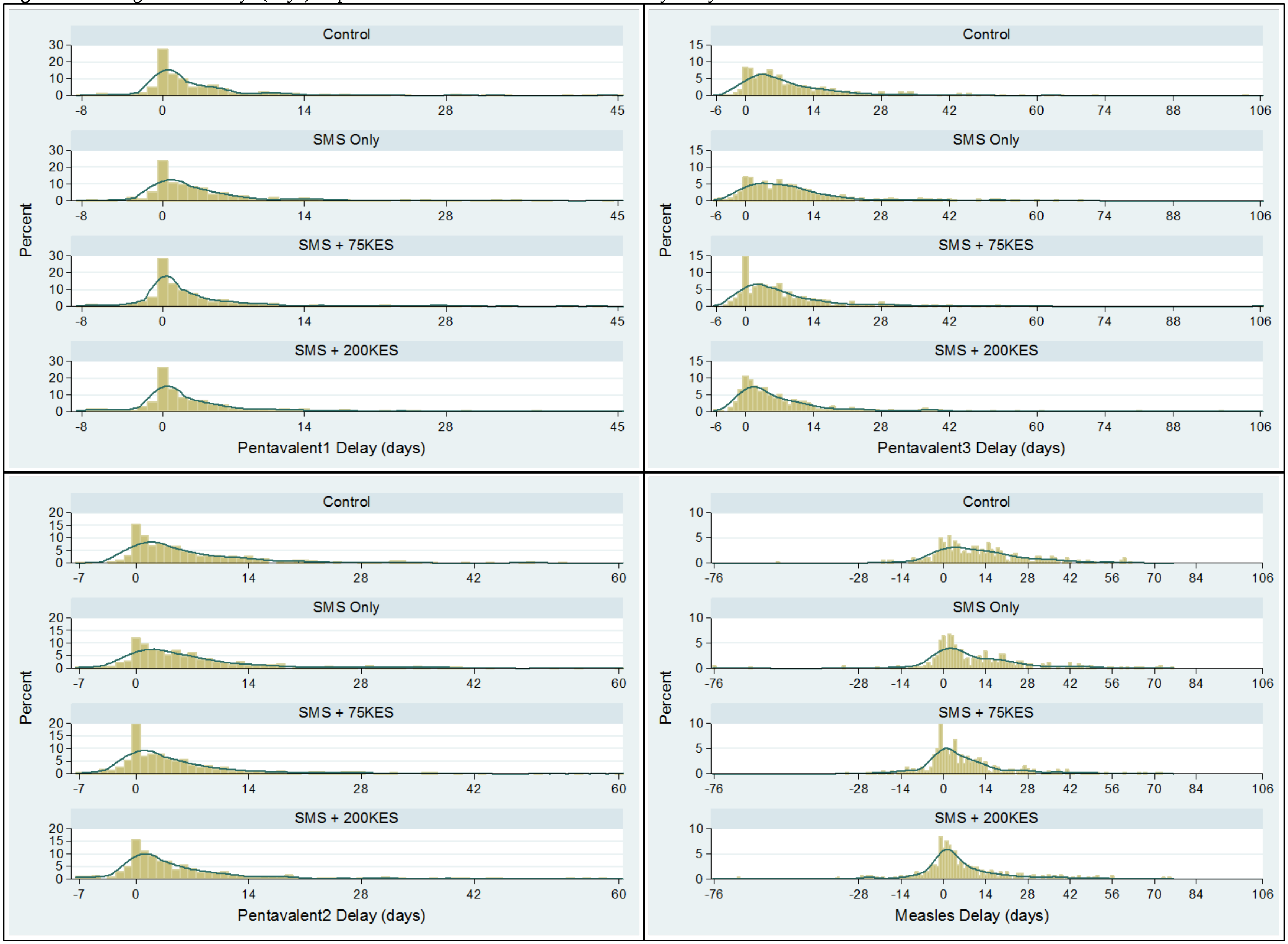

Delays are counted from the recommended age. Children who did not receive vaccination are excluded from analyses. SMS=short message service. KES=Kenyan Shilling. X axis range limits represent 99th percentile of days delayed for each vaccine. Kernel density plot used to smooth the distribution of days delayed

Figure S2. Effect of interventions on time to immunisation

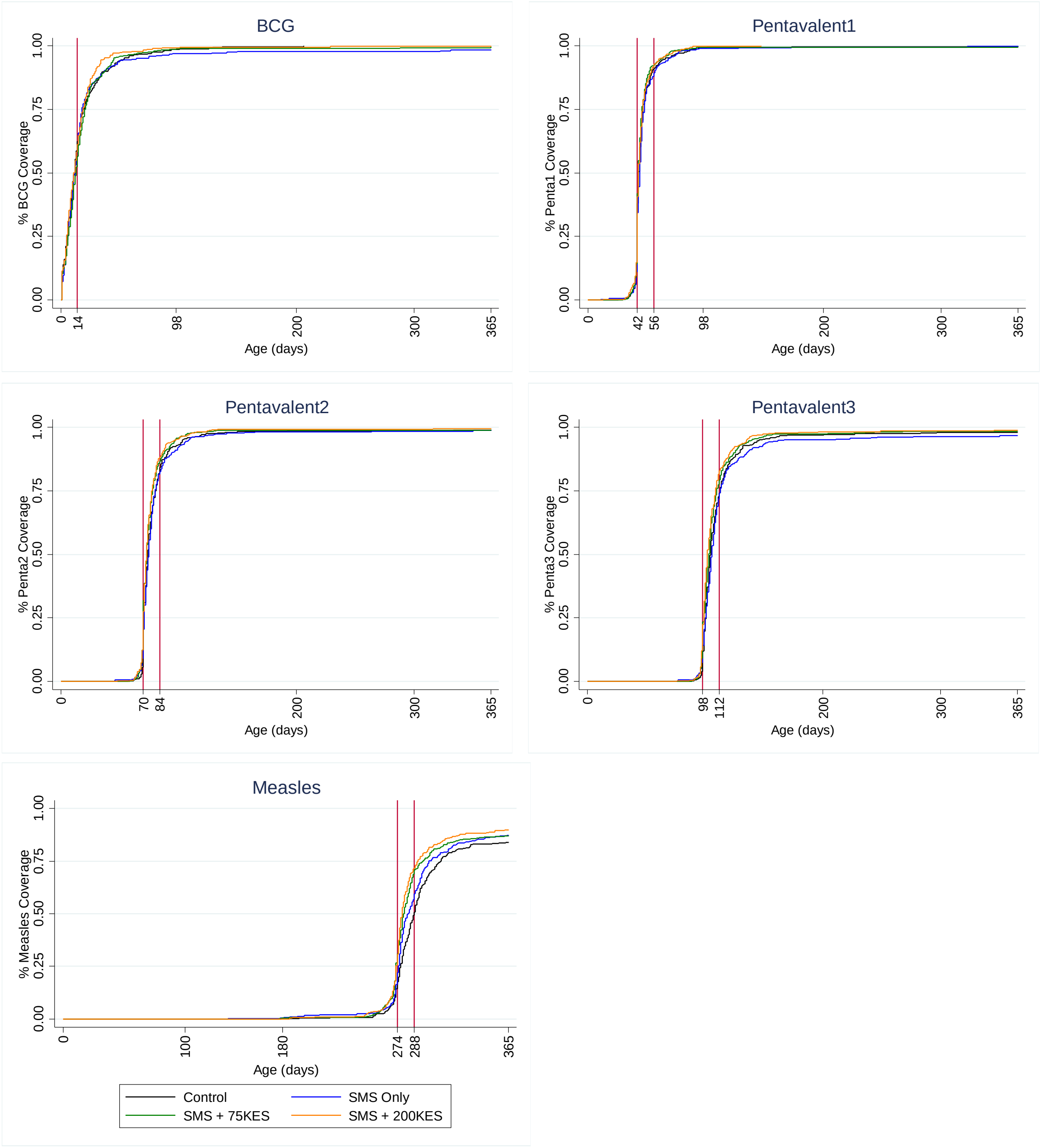

Inverse time to immunisation curves were created using Kaplan-Meier survival analysis. Children were censored at 365 days of age. Vertical red lines indicate the two week window for timely immunisation using the Expanded Progamme on Immunisation schedule. BCG=Bacille Calmette Guerin. FIC=fully immunised child. SMS=short message service. KES=Kenyan Shilling

Table S6. Vaccination timeliness for pentavalent2 and pentavalent3 using an interval-appropriate schedule

	Control (n=360)	SMS* (n=388)	p value†	SMS+75KES* (n=446)	p value†	SMS+200KES* (n=406)	p value†
**Intention-to-treat**							
Penta2	339/360 (94·2%)	368/388 (94·9%)<br>1·01 (0·98–1·04)	0·59	428/446 (96·0%)<br>1·02 (0·99–1·05)	0·16	393/406 (96·8%)<br>1·03 (1·00–1·06)	0·050
Penta3	337/360 (93·6%)	355/388 (91·5%)<br>0·98 (0·94–1·02)	0·27	419/446 (94.0%)<br>1·01 (0·97–1·04)	0·83	388/406 (95·6%)<br>1·02 (0·99–1·06)	0·24
**Per Protocol‡**							
Penta2	339/360 (94·2%)	269/284 (94·7%)<br>1·00 (0·97–1·04)	0·80	325/341 (95·3%)<br>1·01 (0·98–1·05)	0·59	308/341 (97·2%)<br>1·03 (1·00–1·07)	0·068
Penta3	337/360 (93·6%)	287/309 (92·9%)<br>0·99 (0·95–1·03)	0·71	344/366 (94.0%)<br>1·01 (0·97–1·04)	0·85	326/338 (96·5%)<br>1·03 (0·99–1·07)	0·091
**Sent any SMS§**							
Penta2	339/360 (94·2%)	349/366 (95·4%)<br>1·01 (0·98–1·05)	0·42	405/423 (95·7%)<br>1·02 (0·99–1·05)	0·24	375/388 (96·7%)<br>1·03 (1·00–1·06)	0·078
Penta3	337/360 (93·6%)	348/381 (91·3%)<br>0·98 (0·94–1·02)	0·24	415/441 (94.1%)<br>1·01 (0·97–1·04)	0·77	383/401 (95·5%)<br>1·02 (0·99–1·06)	0·25

Data are n (%) and RR (95%CI) for 1600 children with immunisation data recorded on maternal and child health booklet at 12 months. SMS=short message service. KES=Kenyan Shilling. Immunisation timeliness was defined as a child receiving vaccination within 2 weeks of the EPI due date using an interval-appropriate schedule where pentavalent due dates were calculated to be 28 days after previous pentavalent receipt. *Risk Ratios and 95% CI were adjusted to account for correlation within clusters †As compared to control arm. ‡Per protocol is defined as being sent the appropriate number of SMS reminders per vaccine. §Sent any SMS is defined as a caregiver who was sent reminders per protocol or sent at least one SMS.

Table S7. Per protocol analysis of SMS reminders on vaccination timeliness

	Control (n=360)	SMS (n=388)*	p value†	SMS+75KES (n=446)*	p value†	SMS+200KES (n=406)*	p value†
**Per Protocol‡**							
Penta1	328/360 (91·1%)	277/306 (90·5%)<br>0·99 (0·95–1·04)	0·81	338/367 (92·1%)<br>1·01 (0·97–1·06)	0·65	319/341 (93·6%)<br>1·03 (0·98–1·07)	0·24
Penta2	303/360 (84·2%)	247/284 (87·0%)<br>1·03 (0·97–1·10)	0·31	304/341 (89·2%)<br>1·06 (1·00–1·12)	0·045	291/317 (91·8%)<br>1·09 (1·03–1·15)	0·002
Penta3	267/360 (74·2%)	243/309 (78·6%)<br>1·07 (0·97–1·17)	0·18	292/366 (79·8%)<br>1·07 (0·98–1·17)	0·14	287/338 (84·9%)<br>1·14 (1·05–1·25)	0·002
Measles	183/360 (50·8%)	230/386 (59·6%)<br>1·18 (1·01–1·39)	0·037	313/442 (70·8%)<br>1·37 (1·19–1·59)	<0·0001	290/403 (72·0%)<br>1·42 (1·23–1·64)	<0·0001
**Sent any SMS§**							
Penta1	328/360 (91·1%)	324/362 (89·5%)<br>0·99 (0·94–1·03)	0·48	384/415 (92·5%)<br>1·02 (0·97–1·06)	0·49	351/379 (92·6%)<br>1·02 (0·97–1·06)	0·47
Penta2	303/360 (84·2%)	303/366 (82·8%)<br>0·98 (0·92–1·05)	0·61	364/423 (86·1%)<br>1·02 (0·97–1·08)	0·44	341/388 (88·9%)<br>1·04 (0·99–1·11)	0·14
Penta3	267/360 (74·2%)	282/381 (74·0%)<br>1·01 (0·91–1·10)	0·94	350/441 (79·4%)<br>1·07 (0·97–1·16)	0·17	332/401 (82·8%)<br>1·12 (1·02–1·22)	0·012
Measles	183/360 (50·8%)	231/387 (59·7%)<br>1·19 (1·01–1·39)	0·035	316/446 (70·9%)<br>1·37 (1·19–1·59)	<0·0001	292/406 (71·9%)<br>1·42 (1·23–1·64)	<0·0001

Data are n (%) and RR (95%CI) for children with immunisation data recorded on maternal and child health booklet at 12 months and using a per protocol analysis. SMS=short message service. KES=Kenyan Shilling. Immunisation timeliness was defined as a child receiving vaccination within 2 weeks of the EPI due date for vaccines that were reminded or incentivized. *Risk Ratios and 95% CI were calculated using General Estimating Equations with an exchangeable correlation matrix to account for correlation within clusters. †As compared to control arm. ‡Per protocol SMS reminders defined as being sent the appropriate number of reminders per vaccine. §Sent any SMS is defined as a caregiver who was sent reminders per protocol or sent at least one SMS.

Table S8. Sub-group analyses of timely measles vaccination

	Control (n=360)	SMS (n=388)	Stratum-specific RR	p value*	SMS+ 75KES (n=446)	Stratum-specific RR	p value*	SMS+ 200KES (n=406)	Stratum-specific RR	p value*
Overall										
Phone access										
Owns	102/182 (56%)	123/205 (60%)	1.08 (0.90–1.31)	0.167	161/210 (77%)	1.35 (1.15–1.60)	0.745	138/193 (72%)	1.28 (1.07–1.52)	0.081
Shares	81/178 (46%)	108/183 (59%)	1.30 (1.04–1.62)		155/236 (66%)	1.41 (1.15–1.73)		154/213 (72%)	1.58 (1.29–1.92)	
Infant gender										
Male	89/174 (51%)	125/209 (60%)	1.18 (0.96–1.44)	0.949	154/218 (71%)	1.37 (1.13–1.65)	0.910	140/199 (70%)	1.37 (1.13–1.66)	0.605
Female	94/186 (50%)	106/179 (59%)	1.19 (0.97–1.45)		162/228 (71%)	1.38 (1.15–1.66)		152/207 (73%)	1.46 (1.22–1.75)	
SES										
Bottom 40%	58/132 (44%)	70/144 (49%)	1.10 (0.84–1.44)	0.483	118/181 (65%)	1.42 (1.12–1.79)	0.782	118/172 (69%)	1.51 (1.20–1.91)	0.473
Top 60%	125/228 (55%)	161/244 (66%)	1.22 (1.03–1.45)		198/265 (75%)	1.37 (1.17–1.60)		174/234 (74%)	1.38 (1.18–1.62)	
Time to clinic										
≤ 30 minutes	99/202 (49%)	140/225 (62%)	1.30 (1.06–1.59)	0.132	210/293 (72%)	1.46 (1.21–1.76)	0.300	177/255 (69%)	1.43 (1.19–1.74)	0.886
> 30 minutes	84/158 (53%)	91/163 (56%)	1.05 (0.84–1.31)		106/153 (69%)	1.28 (1.04–1.56)		115/151 (76%)	1.41 (1.17–1.71)	
Education										
≤ 7 years	29/83 (35%)	56/97 (58%)	1.66 (1.16–2.36)	0.024	79/124 (64%)	1.80 (1.29–2.52)	0.066	68/107 (64%)	1.82 (1.29–2.55)	0.088
> 7 years	154/277 (56%)	175/291 (60%)	1.09 (0.92–1.28)		237/322 (74%)	1.30 (1.13–1.51)		224/299 (75%)	1.34 (1.16–1.55)	
Maternal age										
≤ 25 years	87/174 (50%)	121/203 (60%)	1.19 (0.98–1.45)	0.960	161/221 (73%)	1.43 (1.18–1.72)	0.629	164/227 (72%)	1.44 (1.19–1.73)	0.803
> 25 years	95/184 (52%)	110/183 (60%)	1.18 (0.96–1.45)		155/223 (70%)	1.35 (1.12–1.61)		128/179 (72%)	1.40 (1.16–1.68)	
Children under 5 yrs in house										
≤ 1	65/122 (53%)	83/133 (62%)	1.14 (0.91–1.43)	0.684	113/146 (77%)	1.41 (1.16–1.72)	0.741	116/157 (74%)	1.36 (1.11–1.66)	0.635
> 1	118/238 (50%)	148/255 (58%)	1.20 (1.00–1.45)		203/300 (68%)	1.36 (1.14–1.62)		176/249 (71%)	1.44 (1.21–1.72)	
District										
Gem	135/285 (47%)	168/301 (56%)	1.18 (0.99–1.42)	0.874	243/354 (69%)	1.42 (1.21–1.68)	0.382	235/325 (72%)	1.52 (1.29–1.79)	0.044
Asembo	48/75 (64%)	63/87 (72%)	1.15 (0.90–1.48)		73/92 (79%)	1.25 (0.99–1.58)		57/81 (70%)	1.12 (0.86–1.44)	

Data are n/N (%) and RR (95%CI) for 1600 children with immunisation data recorded on maternal and child health booklet at 12 months. SMS=short message service. KES=Kenya Shilling. SES=socioeconomic status. *p values obtained from an interaction term between intervention arms and risk factor. †6 missing values.

Table S9.Sub-group analyses of timely pentavalent3 vaccination

	Control (n=360)	SMS (n=388)	Stratum-specific RR	p value*	SMS+ 75KES (n=446)	Stratum-specific RR	p value*	SMS+ 200KES (n=406)	Stratum-specific RR	p value*
Overall										
Phone access										
Owns	143/182 (79%)	160/205 (78%)	1.00 (0.90–1.12)	0.999	174/210 (83%)	1.05 (0.95–1.17)	0.717	163/193 (84%)	1.08 (0.97–1.20)	0.323
Shares	124/178 (70%)	128/183 (70%)	1.00 (0.87–1.16)		180/236 (76%)	1.08 (0.95–1.23)		174/213 (82%)	1.16 (1.03–1.32)	
Infant gender										
Male	129/174 (74%)	153/209 (73%)	0.99 (0.87–1.12)	0.690	173/218 (79%)	1.06 (0.94–1.20)	0.966	161/199 (81%)	1.09 (0.97–1.22)	0.453
Female	138/186 (75%)	135/179 (75%)	1.02 (0.90–1.16)		181/228 (79%)	1.07 (0.95–1.20)		176/207 (85%)	1.15 (1.03–1.28)	
SES										
Bottom 40%	92/132 (70%)	98/144 (68%)	0.98 (0.83–1.15)	0.656	137/181 (76%)	1.07 (0.92–1.24)	0.969	135/172 (78%)	1.12 (0.97–1.29)	0.955
Top 60%	175/228 (77%)	190/244 (78%)	1.02 (0.92–1.13)		217/265 (82%)	1.07 (0.97–1.18)		202/234 (86%)	1.13 (1.02–1.24)	
Time to clinic								75		
≤ 30 minutes	148/202 (73%)	166/225 (74%)	1.02 (0.90–1.16)	0.741	233/293 (80%)	1.08 (0.96–1.21)	0.703	211/255 (83%)	1.13 (1.02–1.27)	0.669
> 30 minutes	119/158 (75%)	122/163 (75%)	0.99 (0.87–1.13)		121/153 (79%)	1.05 (0.92–1.19)		126/151 (83%)	1.10 (0.97–1.24)	
Education										
≤ 7 years	56/83 (67%)	68/97 (70%)	1.04 (0.85–1.27)	0.746	87/124 (70%)	1.03 (0.85–1.25)	0.614	91/107 (85%)	1.26 (1.06–1.49)	0.108
> 7 years	211/277 (76%)	220/291 (76%)	1.00 (0.90–1.11)		267/322 (83%)	1.09 (0.99–1.19)		246/299 (82%)	1.08 (0.8–1.18)	
Maternal age†										
≤ 25 years	133/174 (76%)	150/203 (74%)	0.97 (0.85–1.09)	0.348	181/221 (82%)	1.06 (0.95–1.19)	0.899	188/227 (83%)	1.08 (0.97–1.20)	0.385
> 25 years	133/184 (72%)	137/183 (%)	1.05 (0.92–1.19)		173/223 (78%)	1.07 (0.95–1.21)		149/179 (83%)	1.16 (1.03–1.30)	
Children under 5 yrs in house										
≤ 1	94/122 (77%)	101/133 (76%)	0.99 (0.86–1.14)	0.720	117/146 (80%)	1.03 (0.90–1.18)	0.522	133/157 (85%)	1.09 (0.97–1.24)	0.670
> 1	173/238 (73%)	187/255 (73%)	1.02 (0.91–1.14)		237/300 (79%)	1.09 (0.97–1.21)		204/249 (82%)	1.13 (1.02–1.26)	
District										
Gem	208/285 (73%)	227/301 (75%)	1.03 (0.93–1.15)	0.258	277/354 (78%)	1.07 (0.96–1.18)	0.968	267/325 (82%)	1.12 (1.02–1.24)	0.950
Asembo	59/75 (79%)	61/87 (70%)	0.91 (0.74–1.11)		77/92 (84%)	1.07 (0.90–1.26)		70/81 (86%)	1.11 (0.95–1.31)	

Data are n/N (%) and RR (95%CI) for 1600 children with immunisation data recorded on maternal and child health booklet at 12 months. SMS=short message service. KES=Kenya Shilling. SES=socioeconomic status. *p values obtained from an interaction term between intervention arms and risk factor. †6 missing values.

Table S10: Indirect indicators of health and health utilization

	Control (n=360)	SMS only (n=388)	p value	SMS+75KES (n=446)	p value	SMS+200KES (n=406)	p value
**Received Vitamin A**							
Yes	329 (91%)	337 (87%)		396 (89%)		356 (88%)	
		0·96 (0·89–1·04)	0·29	0·99 (0·92–1·06)	0·74	0·96 (0·89–1·03)	0·27
**Child slept under bed net last night**							
Yes	344 (96%)	362 (93%)		418 (94%)		392 (97%)	
		0·98 (0·94–1·01)	0·17	0·98 (0·95–1·01)	0·25	1·01 (0·98–1·04)	0·53
**Attended HF ≤ 2 weeks (non-vaccination)**							
Yes	90 (25%)	93 (24%)		112 (24%)		102 (24%)	
		0·96 (0·73–1·25)	0·77	1·00 (0·78–1·30)	0·99	1·00 (0·77–1·30)	0·99
**Child hospitalized in past month**							
Yes	15 (4%)	17 (4%)		20 (4%)		18 (4%)	
		1·06 (0·51–2·23)	0·87	1·08 (0·53–2·22)	0·83	1·08 (0·52–2·24)	0·84
**MCH card available at follow-up visit***							
Yes	360/396 (91%)	388/409 (95%)		446/476 (94%)		406/426 (95%)	
		1·04 (1·00–1·09)	0·041	1·03 (0·99–1·07)	0·16	1·05 (1·01–1·09)	0·021
**Caregiver out-migrated†**							
Yes	77/473 (16%)	54/463 (12%)		67/543 (12%)		50/476 (11%)	
		0·73 (0·53–1·02)	0·065	0·75 (0·55–1·02)	0·069	0·62 (0·44–0·87)	0·0057
**Child died before 12 months of age‡**							
Yes	15/411 (4%)	13/422 (3%)		17/493 (3%)		15/441 (3%)	
		0·84 (0·39–1·80)	0·66	0·93 (0·45–1·90)	0·84	0·94 (0·45–1·94)	0·85

Data are n (%) and RR (95%CI). SMS=short messages service. KES=Kenyan Shilling. FIC=fully immunised child. *Data are for 1600 children with MCH booklet and 107 children who verbally reported immunisation at 12 months of age. †Data are for 1600 children with MCH booklet at 12 months, 107 children who verbally reported immunisation history at 12 months, and 248 children who migrated and were lost to follow-up. Children who died (n=60) and withdrew from study (n=3) excluded from sample. Adjusted for maternal age, children under 5 years old in the household, and region ‡Data are for 1600 children with MCH booklet at 12 months, 107 children who verbally reported immunisation history at 12 months, and 60 children who died. Children who out-migrated (n=248) and withdrew from study (n=3) excluded from sample.
